# CD14+ monocytes repress gamma globin expression at early stages of erythropoiesis

**DOI:** 10.1038/s41598-021-81060-7

**Published:** 2021-01-15

**Authors:** Steven Heshusius, Esther Heideveld, Marieke von Lindern, Emile van den Akker

**Affiliations:** 1 Department of Hematopoiesis, Sanquin Research, Plesmanlaan 125, 1066CX, Amsterdam, The Netherlands; 2 Landsteiner Laboratory, Academic University Medical Center, University of Amsterdam, Amsterdam, The Netherlands

**Keywords:** Cell signalling, Transcription, Erythropoiesis

## Abstract

In β-hemoglobinopathies, reactivation of gamma- at the expense of beta-globin is a prominent therapeutic option. Expression of the globin genes is not strictly intrinsically regulated during erythropoiesis, supported by the observation that fetal erythroid cells switch to adult hemoglobin expression when injected in mice. We show cultured erythroblasts are a mix of HbA restrictive and HbA/HbF expressing cells and that the proportion of cells in the latter population depends on the starting material. Cultures started from CD34+ cells contain more HbA/HbF expressing cells compared to erythroblasts cultured from total peripheral blood mononuclear cells (PBMC). Depletion of CD14+ cells from PBMC resulted in higher HbF/HbA percentages. Conversely, CD34+ co-culture with CD14+ cells reduced the HbF/HbA population through cell–cell proximity, indicating that CD14+ actively repressed HbF expression in adult erythroid cultures. RNA-sequencing showed that HbA and HbA/HbF populations contain a limited number of differentially expressed genes, aside from *HBG1/2*. Co-culture of CD14+ cells with sorted uncommitted hematopoietic progenitors and CD34-CD36+ erythroblasts showed that hematopoietic progenitors prior to the hemoglobinized erythroid stages are more readily influenced by CD14+ cells to downregulate expression of *HBG1/2*, suggesting temporal regulation of these genes. This possibly provides a novel therapeutic avenue to develop β-hemoglobinopathies treatments.

## Introduction

Beta-hemoglobinopathies are characterized by mutations within the beta-globin locus, leading to dysfunctional adult hemoglobin (HbA). Attempts to find treatments to these diseases has led to extensive characterization of the key molecular pathways that reactivate gamma-globin (*HBG1/2*) at the expense of beta-globin (*HBB*). Recent progress has focused on BCL11A and LRF1, which are key repressors of HBG1/2 during adult erythropoiesis^1–4^. Different approaches to genetically interfere with the expression of HbF repressors, like BCL11A, are being developed and tested in clinical trials (for review see^5^). However, the requirement for hematopoietic stem cell (HSC) transplantation to introduce the genetically manipulated HSC restricts the scale at which these treatments can be applied. Particularly, because the main endemic countries of these diseases, like African sub-Saharan regions, have limited resources available for transplantation-type treatments. Chemical treatment that targets processes regulating globin expression would allow broad scale application, either in combination with currently used treatments to increase HbF like hydroxyurea, or alone as novel treating entities. The existence of hereditary persistence of hemoglobin (HPFH) in adult erythrocytes as well as the flexibility to re-express HBG1/2 during in vitro erythropoiesis underscore an important degree of flexibility in HBG1/2 regulation. While cultured red blood cells are similar to *ex-vivo* red blood cells in important characteristics, like enucleation, oxygen saturation, deformability and surface protein expression, they frequently express higher levels of fetal hemoglobin (~ 3%) compared to adult erythrocytes^6^. Although the developmental expression pattern of the globin suggests intrinsic regulation, it can also occur extrinsically as fetal-, or induced pluripotent stem cell derived erythroid cells spontaneously switched from fetal-, to adult hemoglobin expression upon injection into irradiated NOD/SCID mice^7^. These data suggest a role for niche cells and signal transduction in extrinsic regulation of globin chains. While in vivo erythropoiesis and hematopoiesis is dependent on niche cells, like central macrophages^8–14^, most erythroid culture models do not take into account the extrinsic signal that initiate from cell–cell interactions. We have previously reported that monocytes/macrophages within the PBMC population positively differentiate into macrophages that resemble erythroid island macrophages that positively influence the hematopoietic stem and progenitor cells leading to increased erythroid outgrowth^15,16^. In contrast to erythroid cells derived from CD34+ cells, those derived from whole blood PBMCs thus arise from HSPCs that are influenced by cells present in PBMCs and by differentiated macrophages originating from PBMC-monocytes. We used this characteristic of the culture system to study regulation of globin subunits by extrinsic factors and demonstrated that PBMC derived erythroid cells have lower HbF compared to CD34+ derived erythroid cells. This is reflected by a smaller fraction of cells expressing gamma globins. The effect depended on cell-proximity between HSPC and the CD14+ fraction from PBMCs, but was absent upon co-culture of already committed erythroblasts with CD14+ cells. In addition, RNA sequencing showed limited differences between cells with and without HbF expression. These data suggest that CD14+ monocytes/macrophages can signal to hematopoietic progenitor cells priming globin expression to *HBB1* at the expense of *HBG1/2*.

## Methods

### Cell sorting

CD14 and CD34 MicroBeads (Miltenyi Biotec; Bergisch Gladbach, Germany) were used for magnetic-activated cell sorting (MACS) of PBMC fractions (manufacturer protocol). Flowcytometric cell sorting was used to sort populations of progenitors based on CD34 and CD36. Sorting after intracellular staining for globin was performed on FACS Aria 4L (BD Biosciences; Oxford, UK).

### Cell cultures

Peripheral blood mononuclear cells (PBMCs) were obtained according to the Declaration of Helsinki (seventh revision, 2013). Written informed consent to extract and use human material (peripheral blood) as part of regular donations was obtained with approval of the local medical ethics committee (MEC, Sanquin). Experimental protocols were approved by Sanquin (MEC). Human PBMC from whole blood were purified by density separation using Ficoll-Paque (manufacturer protocol). CD34+ cells were isolated from PBMCs by MACS. Both were cultured in a three-phase liquid erythroid culture system as previously described^6^. Low iron experiments contained 100 mg/ml holotransferrin one third of the concentration normally used throughout culture.

### Co-culture

CD34 and CD14 fractions were put in co-culture at the ratios indicated in the legends, (ratio in blood is ~ 1:100 CD34:CD14). Trans-well co-cultures were performed by seeding CD14+ cells at the bottom and CD34+ in the trans-well (0.4 µm polyester membrane, Corning; NY, USA), cultured for 8 days and differentiated for two days to allow hemoglobinization as described before^6^. For co-cultures with sorted CD34CD36 fractions, freshly isolated CD34+ cells were cultured for three days, sorted and co-cultured for an additional 5 days.

### Flowcytometry

Cells were washed twice with PBS and incubated in FACS-buffer (1% bovine serum albumin) with surface antibodies against CD71-VB405 (Miltenyi Biotec; 1:200), CD235-PE (Acris; 1:2500), CD14-APC (Miltenyi Biotec; 1:50), CD16-PE (BD Biosciences; 1:80); CD36-FITC (Pelicluster, Amsterdam, The Netherlands; 1:100); CD34-APC (IQ products, Groningen, the Netherlands; 1;10). Isotype controls were IgG1k-FITC (biolegends), IgG1-PE (Diaclone); IgG1k-APC (eBioscience). For hemoglobin staining on erythrocytes cells were fixed with 0.025% glutaraldehyde (sigma-Aldrich) and 0.5% paraformaldehyde (Signa-Aldrich) in PBS and permeabilized with 0.5% NP40 (Sigma-Aldrich) prior to staining HbA-PE (Santa Cruz; 1:1000) and HbF-APC (Invitrogen; 1:1000).

### HPLC

1*10^7^ cells were collected and analysed for Hb isoform expression by high-performance cation-exchange liquid chromatography (HPLC) on Waters Alliance 2690 equipment as previously described^17^.

### QPCR

Samples of 48 h differentiated erythroblasts were collected from three individual donors. RNA was isolated using TRIzol RNA isolation reagent (ThermoFisher Scientific).

### RNA sequencing and data analysis

A technique previously described by Nicolet et al. was used to reverse crosslinking after intracellular globin staining, prior to sequence library preparation^18^. Sequencing libraries were prepared using Trizol RNA isolation, cDNA amplification and rRNA depletion using HyperPrep Kit with RiboErase (KAPA Biosystems, Pleasanton, CA, USA) as described by manufactures. Samples were sequenced to a depth 30 × 10^6^ × 75 bp paired end reads. RNA-seq reads were mapped to GRCh38v85 using STAR. Lowly expressed genes (1 count per million mapped reads in at least two samples) were filtered prior to differential expression analysis in EdgeR, testing differential expression in a paired design with a quasi-log-likelihood F-test.

### Real-time quantitative PCR

cDNA was synthesized with the QuantiTect reverse transcription kit (QIAGEN, #205313) according to the manufacturers’ instructions and Quantitative PCR was performed on a StepOnePlus Real-Time PCR system (ThermoFisher Scientific, #4376600) using Power SYBR green master mix (ThermoFisher Scientific, #4367659; 1 μM primers; final volume 20 μl; cycles: 10 min 95 °C; 40 cycles: 15 s at 95 °C and 1 min at 60 °C). Ct-values were normalized against housekeeping genes 18S and HPRT. Graphpad Prism V7.04 (GraphPad Software) was used for statistic testing and visualization of fold-change mRNA expression. RT-PCR-Primers used (5′–3′): *HBB*fw; ACAGCCACCACTTTCTGAT; *HBB*rv, AGCTGCACTGTGACAAGCTG; *HBG1/2*fw, AAACGGTCACCAGCACATTT; *HBG1/2*rv, GAAGGTGCTGACTTCCTTGG; *18S*fw, CACGGCCGGTACAGTGAAAC; *18S*rv, AGAGGAGCGAGCGACCAA; *G3PH*fw, CATCACGCCACAGTTTCC; *G3PH*rv, TCCCATCACCATCTTCCA.

## Results

### CD34+ derived erythroblasts express significantly higher levels of HbF compared to PBMC-derived erythroblasts

**Figure 1:**
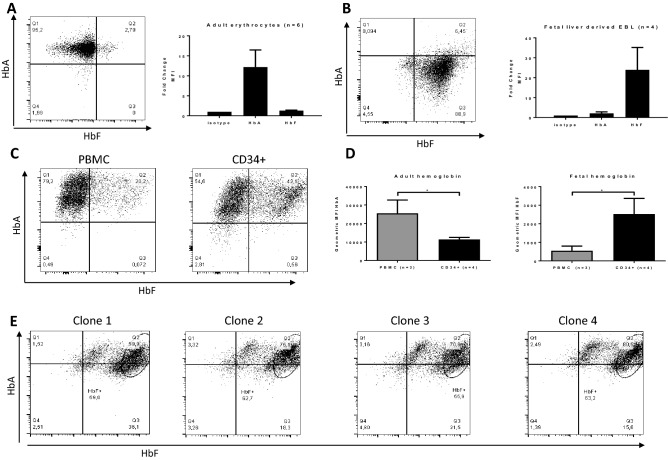
Expression of HbF and HbA is dependent on erythroblasts culture starting material. Erythrocytes from human adult peripheral blood (**A**; n = 6) or human fetal liver (**B**; n = 4) were stained with anti-HBB1 (y-axis) and anti-HBG1/2 (x-axis). Representative dot plots are shown and the histograms indicate the mean fluorescent intensity of the complete globin expressing population. (**C**) erythroblasts were cultured from PBMC or from CD34+ cells isolated from PBMC. Expression of HBB1 and HBG1/2 was measured as indicated in (A). Representative dot plots are shown. (**D**) The histograms indicate the mean fluorescent intensity of the complete globin expressing population (PBMC, n = 3 and CD34+ cells, n = 4; *p < 0.05 Student’s T-test). (**E**) CD34+ cells were single cell sorted and cultured as indicated in material and methods. The dot plots represent anti-HBB1 (y-axis) and anti-HBG1/2 (x-axis) of four different clonal CD34+ -single-cell-derived erythroblast populations.

In the majority of adults, fetal hemoglobin (HbF) makes up less than 1% of the total hemoglobin content in erythrocytes. However, erythrocytes of individuals with hereditary persistence of fetal hemoglobin (HPFH), and also in vitro cultured adult erythroid cells, may express HbF at levels higher than 5% as determined by HPLC^6^. HPLC measures bulk hemoglobin and cannot discriminate between increased numbers of cells expressing gamma globin or increased gamma expression in the fraction of cells that already express HbF. To this end, we analyzed hemoglobin expression in *ex-vivo* and cultured erythroid cells by flow cytometry. Upon intracellular staining for beta globin and gamma globin, components of HbA and HbF respectively, adult erythrocytes showed mostly HbA, with a fraction of cells positive for HbF (Fig. 1A). Erythroblasts from human fetal liver predominantly showed HbF expression, indicating that the assay is specific for the two globin types (Fig. 1B). In contrast to adult and fetal *ex-vivo* erythroid cells, cultured adult erythroid cells showed two distinct populations; one expressing HbA and one expressing both HbF and HbA (Fig. 1C). No cells expressing only HbF are found. Of note, erythroid cells cultured from cord blood are significantly enriched in HbF/HbA expressing cells, reflecting the ongoing globin switching occurring at this developmental stage (supplemental Fig. 1A). The increase in HbF expression in adult cultures was dependent on the starting source for erythroid cultures. Erythroid cultures initiated from total adult peripheral blood mononuclear cells (PBMC) showed a significantly lower HbF expression and higher HbA expression compared to cultures that were started from purified CD34+ cells from PBMC (Fig. 1C, D). The increase in HbF and the linked decrease in HbA suggests an active regulation of globin chain expression. HPLC measurements confirmed that cultures from PBMC contained 1–3% HbF compared to > 5% HbF in cultures started from selected CD34+ cells (supplemental Fig. 1B. CD34+ cells are a heterogeneous population of hematopoietic progenitors at various stages that upon culture may result in two different populations of HbA and HbA/HbF expressing cells. Thus, we tested if the two populations (HbA or HbA/HbF) can arise from one single CD34+ cell or whether the ability to express HbF is segregated at the progenitor level. Single cell sorted adult CD34+ cells were differentiated to erythroid cells. The single cell cultures still showed two populations, one expressing high HbF and one expressing low to no HbF ruling against the presence of two different parental hematopoietic progenitors. Cultures from different clones showed between 56 and 63% of cells in the HbF high population (Fig. 1E), which was significantly higher compared to non-single cell derived CD34+ cultures. The presence of two populations in all clones shows that erythroid cells derived from a single CD34+ cells can express different hemoglobin expression profiles. The data also indicated that in a selection of cells the HBG1/2 remains silent. Of note, to obtain enough cells to perform flow cytometry for HbA and HbF the CD34+ single cells were cultured for a minimum of 25 days.

### CD14+ monocyte/macrophages from PBMC reduce HbF expression in erythroid cultures

**Figure 2:**
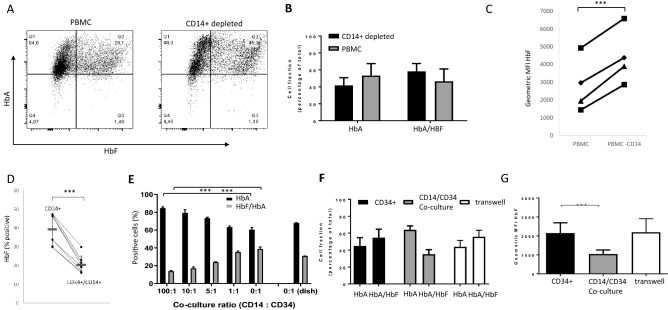
CD14+ monocytes promote repression of HbF expression in erythroblasts. (**A**) PBMC and PBMCs depleted for CD14+ cells were cultured to obtain hemoglobinized erythroblasts as indicated in material and methods. The expression of HBB1 and HBG1/2 was assessed by flow cytometry. Dot plots show representative erythroblasts cultured from PBMC (left) or PBMC depleted for CD14+ cells (right; anti-HBB1, y-axis; antiHBG1/2, x-axis). (**B**) Bar graph shows the percentage of events in Q1 (HbA cells) and Q2 (HbA/HbF cells) from (**A**) for erythroblasts derived from PBMC (grey bars) or from CD14+ depleted PBMC (black; n = 3). (**C**) Mean fluorescence intensity of HBG1/2 from the data presented in; N = 4; paired student TTEST; *** = p < 0.01 (**A**). (**D**) CD34+ cells and CD14+ cells were purified from PBMC and co-cultured or not as indicated in the graph. The plot depicts the HbF percentage in the different culture conditions and lines indicate the paired samples (N = 4 donors in duplicate; a paired T-test, *** = p < 0.01). (**E**) CD34+ and CD14+ cells were purified from PBMC and co-cultured using specific ratios of CD14+ cells to CD34+ cells as indicated on the x-axis (CD14+ :CD34 +). Bar graphs represent the percentage of cells that are HBB1 positive (Black, HbA) or HBB1/HBG1/2 double positive (grey, HbA/HbF) as determined by flow cytometry (supplemental Fig. 3; n = 3; student TTEST p < 0.01). (**F**, **G**) CD34+ and CD14+ cells were purified from PBMC as indicated in material and methods. Cells were co-cultured using 100:1 CD14+ to CD34+ ratio in full contact (grey bars), separated by transwells (white bars). The black bars represent CD34+ cultured without CD14+ cells as a control. Bar graphs in (**F**) represent the percentage of cells that are HBB1 positive (HbA) or HBB1/HBG1/2 double positive (HbA/HbF) as determined by flow cytometry (n = 3). Bar graph in (**G**) depicts the geometric mean fluorescence of HBG1/2 expression (HBF) as measured by flow cytometry (n = 3, ***p < 0.01; Student’s T-test).

Erythroid differentiation in the bone marrow occurs on erythroblast islands, structures of erythroid progenitors surrounding a central macrophage^8,9^. Defects in the interaction between macrophages and erythroid cells have been reported to result in erythropoiesis defects^14,19^. PBMC cultures contain significant amounts of cells capable of providing support and/or interactions with CD34+ , erythroid progenitors and erythroblasts. In fact, 14% of PBMCs are CD14+ monocytes that can differentiate to erythropoiesis supporting macrophages in our culture medium^15,20^. CD14+-derived macrophages from PBMC interact with, and support HSPC^15,21,22^. To test if a similar interaction was responsible for the repression of HbF, PBMCs were depleted for CD14+ cells. Differentiating erythroblast from CD14+ depleted PBMCs showed a significantly increased frequency of HbA/HbF expressing cells and a higher HbF mean fluorescent intensity (MFI) compared to erythroid cultures derived from total PBMCs (Fig. 2A–C). The increased fetal hemoglobin expression did not result from overall lower hemoglobinization (supplemental Fig. 2). Of note, cultures that received suboptimal levels of holo-transferrin did show reduced hemoglobinization (supplemental Fig. 2). Next, we asked if CD14+ alone would be sufficient to reduce HbF in CD34+ cultures. The CD34+ fraction and the CD14+ fraction were isolated from buffy coats and co-cultured. Co-culture of CD34+ cells with CD14+ cells lead to a decrease in HbF expression compared to CD34+ alone (Fig. 2D and supplemental Fig. 3A), which occurred in a dose dependent manner (Fig. 2E, supplemental Fig. 3B). To test if reduced HbF expression required direct contact or resulted from a secreted factor, CD34+ were differentiated to erythroid cells in a transwell co-culture setup. Although co-culture in direct contact with CD14+ positive cells again reduced HbF expression in erythroid cultures, erythroblasts co-cultured with CD14+ cells in a transwell setting did not reduce HbF expression (Fig. 2F, G). This suggests that HbF repression in culture requires cell–cell proximity of CD14+ and erythroid progenitor cells.

**Figure 3:**
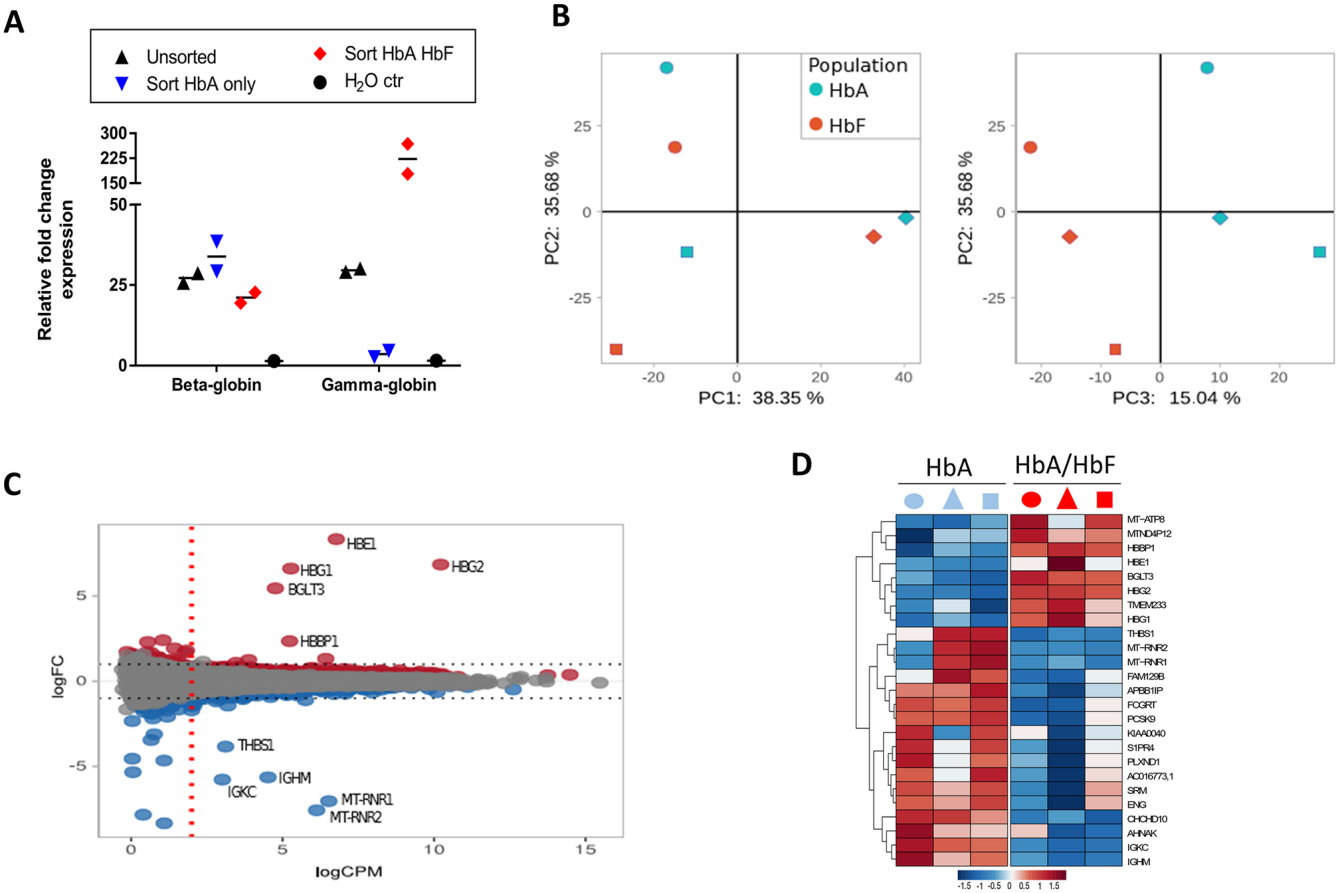
The RNA expression profile between sorted HbA and HbA/HbF expressing erythroblasts is highly similar. Erythroblasts cultured from three independent donors were stained for HBB1 and HBG1/2 and the HBB1 and HBB1/HBG1/2 expressing erythroblasts were FACS-sorted after which RNA was isolated and subjected to RNA-sequencing as indicated in material and methods. (**A**) RT-qPCR using primers for beta-globin (HBB1) and gamma-globin (HBG1/2) on mRNA isolated from HbA (blue) and HbA/HbF (red) sorted populations, unsorted (black) and water control (black circles) as indicated. The relative fold change expression to the water control is depicted. (**B**) principle component analysis showing the variation between the sorted populations in the three first components. (**C**) RNA sequencing expression analysis depicted as log2 fold change (y-axis) against average count per million reads per gene (CPM, x-axis). Differentially expressed genes crossing the threshold of p < 0.01 are indicated in red for upregulated and blue for downregulated (dark spots represent unchanged RNAs). The blue lines indicate the log2 fold change cut-off (CPM), − 1 < gene > 1 and the red line indicates the CPM cutoff (> 2). (**D**) Heatmap depicting z-scores of the 25 differentially expressed RNAs clustered (rows and columns) using one-minus-Pearson-correlation with average linkage.

### The transcriptomes of HbA/HbF co-expressing compared to HbA only expressing cells is highly similar

The presence of HbF/HbA co-expressing cells and HbA single expressing cells in culture, combined with the observation that the balance between these populations can be regulated through specific interactions with monocyte/macrophage populations suggests that potential globin regulators may be differentially regulated upon comparing these two erythroid populations. To find such potential targets the transcriptome of HbF/HbA co-expressing and HbA expressing populations cultured from adult PBMCs was assessed by RNA sequencing of the erythroblast stage (CD71+/CD235+; supplemental Fig. 4A). RT-PCR on the RNA isolated from these population indicated beta globin expression in HbA or HbF/HbA cells, however beta-globin levels were ~ fourfold lower in HbF/HbA cells (Fig. 3A). As expected, gamma-globin levels were nihil in HbA only and high in HbF/HbF cells. Principle component analysis of the corresponding, normalized transcriptome data revealed that the first two components (45% of the variation) separated samples by donor, while the third component (15% of the variation), separated the HbF/HbA sorted population from the HbA population (Fig. 3B. Interestingly, out of over 16,000 distinct RNA species detected only a surprisingly small number of 25 genes (8 up and 17 down in HbF/HbA vs HbA expressing cells) were differentially expressed with at least a two-fold difference between the two sorted populations (false discovery rate (FDR) < 0.01; fold change: − 2 < FC > 2; Log count per million CPM > 2; Fig. 3B–D; Table 1). HBG1 and HBG2, were the most differentially expressed between the two populations conforming the sorting results by HPLC and flow cytometry in Fig. 1 (supplemental Fig. 4A). Among the 8 RNAs upregulated in HbF/HbA cells, 5 were located within the beta globin locus. Besides *HBG1/2*, these were embryonic epsilon globin chain (*HBE1*), and the non-coding RNAs *BGLT3* and *HBBP1*, both non-coding RNAs were significantly higher expressed. In addition, two mitochondrial RNAs and one transmembrane protein TMEM233 were upregulated (Table 1). GO analysis indicates oxygen carrying/binding (upregulated RNA’s) and transforming growth factor binding (down regulated RNA’s) as enriched GO terms (data not shown). Among the genes that are expressed at lower levels in HbF/HbA cells compared to HbA only cells are *Endoglin* (*ENG*), *Thrombosondin* (*THBS1*), *Pleckstrin* (*PLEK*), *Plexin D1* (*PLXND1*), *Rap1-interacting adapter molecule* (*RIAM*/ *APBB1IP*) and *AHNAK,* encoding a large intracellular scaffold protein, which may all alter response to environmental factors for instance signal transduction by TGFβ (transforming growth factor beta) family members. Similarly, reduced expression of the *sphingosine-1-phosphate receptor 4* (*S1PR4*) may alter response to the environment. Several transcriptional regulators are known that control or influence the expression of globin subunits. However, known globin regulators, BCL11A, KLF1, ZBTB7A and SOX6 were not found to be differentially expressed (Supplemental Fig. 4C). Adding to this, we do not observe a reduction in KLF1 target genes (e.g. CD44, BCAM, CARM1, BCL11A; Supplemental Fig. 4C). Moreover, among the deregulated RNA’s no other transcriptional regulator was identified.

**Figure 4:**
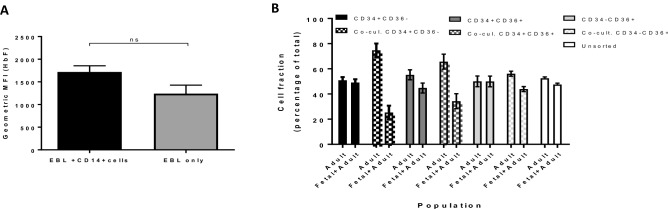
Inhibition of HbF expression by CD14+ cells occurs early during hematopoiesis/erythropoiesis. (**A**) Erythroblasts (CD71+/CD235low) were cultured with and without PBMC-isolated CD14+ cells as indicated in material and methods. Bar graph shows the mean fluorescent intensity of gamma globin expression as measured by flow cytometry. (**B**) Hematopoietic progenitors were sorted based on their expression of CD34 and CD36 expression. In short, CD34+CD36− represent hematopoietic stem and progenitors cells, CD34+CD36+ cells are committed to a megakaryoid/erythroid fate but no myeloid fate and CD34−CD36+ cells are pro-erythroblasts^15^. Cells were cultured in presence (solid bars) or absence of PBMC-isolated CD14+ cells (striped bars). The percentage of HbA and HbA/HbF cells was measured by flow cytometry and indicated on the y-axis. Note that the reduction of HbF/HbA populations by CD14+ cells is proportional to the immaturity of the progenitors (N = 3).

**Table 1:** Up and down regulated genes in HbF/HbA expressing cells versus HbA only expressing cells.

Gene name	logFC	logCPM	F	PValue	FDR
IGHM	− 5.63562	4.517326	774.7776	1.16E−17	4.85E−14
IGKC	− 5.771	3.010395	353.4626	2.52E−14	8.45E−11
THBS1	− 3.83388	3.116554	259.4671	4.82E−13	1.16E−09
MT-RNR1	− 7.56982	6.122425	217.4913	2.53E−12	5.01E−09
MT-RNR2	− 7.0361	6.52396	216.121	2.68E−12	5.01E−09
ENG	− 1.10868	5.75179	53.62633	4.02E−07	0.000181
FCGRT	− 1.42587	3.190215	49.07273	7.76E−07	0.000241
PLXND1	− 1.04451	3.690973	33.31091	1.13E−05	0.001075
AHNAK	− 1.03942	2.854409	32.673	1.28E−05	0.001143
S1PR4	− 1.02594	3.000601	31.28129	1.69E−05	0.001367
CHCHD10	− 1.09501	2.231917	25.20431	6.26E−05	0.002818
SRM	− 1.18123	2.815445	24.4092	7.54E−05	0.003203
FAM129B	− 1.45553	2.062975	24.279	7.77E−05	0.003271
APBB1IP	− 1.02367	2.427376	23.90906	8.48E−05	0.003424
KIAA0040	− 1.08592	2.147614	22.67123	0.000114	0.004113
PCSK9	− 1.04046	2.166769	20.12631	0.000218	0.006204
AC016773.1	− 1.01497	2.064593	18.45859	0.000341	0.008104
HBG2	6.853673	10.21744	3839.172	1.22E−24	2.04E−20
HBG1	6.617616	5.271699	1226.88	1.19E−19	9.95E−16
BGLT3	5.457426	4.761285	783.0324	1.04E−17	4.85E−14
HBBP1	2.356147	5.231101	282.5109	2.15E−13	6.02E−10
HBE1	8.343875	6.773354	194.2372	7.24E−12	1.21E−08
MT-ATP8	1.323711	6.422302	117.2473	6.81E−10	1.04E−06
TMEM233	1.253602	3.878552	88.31424	7.67E−09	8.77E−06
MTND4P12	1.085315	3.462607	21.83769	0.000141	0.004639

### Repression of HbF by CD14+ cells occurs before erythroblasts stage

As the number of differentially expressed genes is surprisingly low between HbA/HbF and HbA sorted populations and cannot be attributed to RNA expression levels of known or novel transcriptional regulators, we hypothesized that posttranscriptional control mechanisms regulate the frequency of HbF/HbA cells, and/or (*ii*) globin subunit regulation occurred before the hemoglobinized CD71+/CD235+ erythroblast stage analyzed and that the molecular mechanism driving this differential globin expression may already be downregulated at the hemoglobinized erythroblast stages. The alternative explanation would fit with high HbF in the cultures from single cell CD34+ in Fig. 1E, if this were to result from prolonged expansion of the earliest progenitor stage. Indeed, CD34+ hematopoietic stem and progenitor cells (HSPC) are a mix of hematopoietic cells at different stages of differentiation from hematopoietic stem cells to more committed lineage progenitors^15^. Lineage specification progresses from CD34+ CD36- hematopoietic stem and progenitor cells to CD34+ CD36+ megakaryoid/erythroid common progenitors to CD34-CD36+ erythroblasts^15,23^. Co-cultures with these fractions and the CD14+ cells were assessed to determine the stage at which the repression occurred. Co-culture of CD34-CD36+ erythroblasts with CD14+ monocytes did not decrease HBG1/2 expression. This supports the RNA-sequencing data results that the regulation of HBG1/2 in erythroid cultures by CD14+ cells does not occur at the committed lineage restricted (pro)erythroblast stage (Fig. 4A). In contrast, CD14+ cells were able to repress *HBG1/2* expression in CD34+CD36−/+ progenitors with the biggest repression occurring in the early CD34+CD36− co-cultures with CD14+ cells (Fig. 4B). Of note, repression of HbF lead to a concomitant increase in HbA. The data indicate that regulation of globin genes by CD14+ cells can occur early during hematopoiesis before the commitment to erythroid restricted progenitors.

## Discussion

Hematopoietic stem cell differentiation to erythroid cells occurs within the bone marrow. This process depends on interactions with specific cells present within the different bone marrow niches. Distinctive supporting macrophages can be identified both in the hematopoietic stem and progenitor cell niche as well as within the erythroid niche, where they can provide signals to the differentiating hematopoietic cells^8,9,24–26^. Reminiscent to these niche functions, PBMC-isolated CD14+ monocyte-derived macrophages were found to both support survival of HSPC and recapitulate central macrophage functions^15,20,21^. Here we find that the CD14+ monocyte-derived macrophages alter the balance between gamma and beta globin gene expression in erythroid cultures. Specifically, we find that gamma-globin down regulation occurs prior to the erythropoietin, stem cell factor and glucocorticoid responsive non-hemoglobinized committed erythroblast stage, defined as CD71^high^/CD235^low/+^^6^. In fact, the highest repression was observed at the earliest CD34+CD36− hematopoietic stem and progenitor population, suggesting that erythroid specific factors like KLF1 are not involved in this regulation. Indeed, KLF1 was not deregulated between hemoglobinized erythroblasts expressing HbA-only or HbA/HbF. Moreover, the regular suspects of globin regulation, e.g. SOX6, BCL11A and LRF were not differentially expressed at the erythroid committed erythroblast stage. However, as some of these regulators (e.g. BCL11A) are expressed and functional at the HSPC stage or even in HSCs^27–29^, the data does not rule out a role for these factors to regulate HbA/HbF expression in early hematopoietic progenitors. The data suggests a novel concept that globin expression induced later during erythroid differentiation can be regulated at these early HSPC progenitor stages. This would fit with the proposed flexibility in globin-chain expression observed by Kobari et al., who have reported that SCD-iPSC derived erythroid progenitor cells that in vitro express fetal hemoglobin (HbF) will convert to adult beta globin (HbA) expression containing the mutated adult beta sickle cell HBS peak in HPLC upon injecting in vivo, revert to producing beta-globin expressing erythrocytes upon injection into NOD-SCID mice^7^. In addition, Singh et al. have shown that the gamma-globin promoter was highly methylated in the earliest stage of hematopoietic stem progenitor cells (CD34( +)CD36(−)) and that methylation progressively decreased as HSPC differentiation progressed in sorted adult bone marrow progenitors to committed erythroblasts^30^. Although, we did not look at the methylation status of the promoters in our erythroid cultures, combined with the flexibility in globin chain expression it substantiates a hypothesis where the niche can instruct erythropoiesis to express specific globin subunits in a process that involves epigenetic modifications at the earliest differentiation stages of erythropoiesis. In line with the role of a niche cell, the CD14+-derived macrophages from PBMCs regulate globin expression through cell–cell contact. We and others have previously shown that these PBMC-CD14-derived CD163/CD169+ macrophages resemble and function like erythroid island central macrophages^20,21^. We propose that also macrophages within the bone marrow niche may similarly exert signaling to regulate globin expression. We acknowledge that the ratio between the macrophages and the erythroid precursors is off and in favor of macrophages, whereas in the niche one macrophage is surrounded by > 10 erythroid cells. However, as a clear dose response curve of macrophages on the repression of HbF is found, this clearly indicates that signaling influences specific globin subunit expression. Importantly, here we use a macrophage co-culture system with erythroid precursors recapitulating only one component of the hematopoietic/erythroid niche in vitro and hence a simplification of processes occurring within the bone marrow. Despite these considerations, the data indicate that hematopoietic progenitors can be influenced by signaling to control globin expression and provides a possible mechanism through which the bone marrow niche specifically produces beta globin expressing erythroid cells. The precise identity of these signals and the induced signal transduction in erythroid progenitors remains unknown but are important to elucidate. Mapping the signal transduction induced by macrophages on erythroid/hematopoietic progenitors may link to the well-known regulators of the globin genes or may uncover novel pathways. These pathways could potentially be blocked to increase gamma expression providing novel therapeutic options to increase HbF in sickle cell or beta-thalassemia either on its own or in combination with other (HbF-inducing) therapy.

## Supplementary information

Supplementary information.
