# Supplementary material for: CD14+ monocytes repress gamma globin expression at early stages of erythropoiesis
Source: Sci Rep. 2021 Jan 15;11:1507. doi: 10.1038/s41598-021-81060-7 (PMC7810836; doi:10.1038/s41598-021-81060-7)
Supplement: Supplementary file 1 — Supplementary information. [file 41598_2021_81060_MOESM1_ESM.pdf]

**Supplementary material**

**CD14+ monocytes repress gamma globin expression at early stages of erythropoiesis**

Steven Heshusius[1], Esther Heideveld[1], Marieke von Lindern[1] and Emile van den Akker*[1]

[1]Sanquin Research, Department of Hematopoiesis, Amsterdam, The Netherlands, and Landsteiner Laboratory, Academic University Medical Center, University of Amsterdam, Amsterdam, The Netherlands

*Correspondence: Emile van den Akker, PhD, Sanquin Research, Department of Hematopoiesis, Plesmanlaan 125, 1066CX Amsterdam, The Netherlands. Telephone: 0031-(0)631010473; Fax: 0031-(0)205123474; e-mail: e.vandenakker@sanquin.nl

Supplemental figures: 4

**Supplemental legends**

*Supplemental figure 1. Globin subunit and hemoglobin measurements in cord blood and through HPLC.* **A)** cord blood derived CD34+ cells (n=5) were cultured to hemoglobinized erythroblasts as described in material and HBB1 and HBG1/2 were measured. Upper dot plots display HBB1 staining (y-axis, HbA) and HBG1/2 stainings (x-axis, HbF). Lower dot plots display CD71 (Y-axis) and CD235 (x-axis) stainings, indicating erythroid differentiation progression. **B)** HPLC to fractionate the different forms of hemoglobin of CD34+ derived erythroblasts was performed as described in material and methods. The graphs show the absorbance at 415nm (y-axis) as a function of the retention time in minutes (x-axis). The identity of the different peaks is indicated with the blue arrow indicating the retention time of HbF with the percentage of HbF calculated as the fraction of the area under the curve of the HbF peak of the total area under the curve of all peaks.

*Supplemental figure 2. Erythroid cells cultured from PBMC that were CD14+ cell depleted are unchanged in total hemoglobinization.* PBMCs or PBMCs depleted for CD14+ cells were cultured to hemoglobinized erythroblasts as indicated material and methods. In addition, PBMCs were cultured in low holotransferrin conditions (low iron,

material and methods). Hemoglobin per cell was determined by performing o-dianosine benzidine assay to quantify the total amount of heme. No difference in the amount of heme was observed between PBMC (grey bars) and PBMC-CD14 (black bars). Lowering the total amount of available iron (through holo-transferrin) did lower total heme (PBMC-white bars). N=3, p<0.01 Student's T-test.

*Supplemental figure 3. Increasing the ratio of CD14+ cells to CD34+ cells in co-culture results in a dose dependent effect of HbF repression.* CD14+ cells and CD34+ cells from three different healthy donors were isolated and co-cultured in a fixed ratio (A, 4 donors in duplicate) or in the ratios indicated (B, 3 donors). The dot plots represent globin expression measured using anti-HBB1 (HbA, y-axis) and anti-HBG1/2 (HbF, x-axis) belonging to main figure 2D (A1) or figure 2E (B). A2 represent CD71/CD235 stainings belonging to figure 2D, note that the cultures are all in a comparable erythroid differentiation state.

*Supplemental figure 4. Comparing the RNA expression profiles between HbA and HbA/HbF expressing cells is highly similar.* **A)** CD34+ cells from three healthy donors were cultured to hemoglobinized erythroblasts as indicated in material and methods. The upper dot plots represent HBB1 (HbA, y-axis) as a function of HBG1/2 (HbF, y-axis) and the lower blots indicate the differentiation progression as determined by stainings for CD71 (y-axis) and CD235 (x-axis). The HbA and the HbA/HbF expressing cells were sorted and subjected to RNA-sequencing as described in material and methods. **B)** Integrative genomics viewer (IGV) [james, 2017]) showing the mapping of RNA-sequencing reads (y-axis) onto the beta globin locus (x-axis). Note that the HbA/HbF sorted cells express high levels of HBG1/2 compared to HbA sorted cells. Lower IGV zoom in displays the HBG1 and HBG2 mRNA signal and potential read through into the neighboring BGLT3 non-coding-RNA gene in the HbA/HbF sorted cells but not in the HbA sorted cells (n=3). **C)** Heatmap displaying a Pearson clustering of z-scores calculated from CPM for the indicated selection of globin locus regulators (y-axis). Note that the HbA and HbA/HbF populations do not segregate (x-axis) showing that variations within the expression of these genes does not explain the higher HbF expression in the HbA/HbF sorted cells.

# Supplemental figure 1

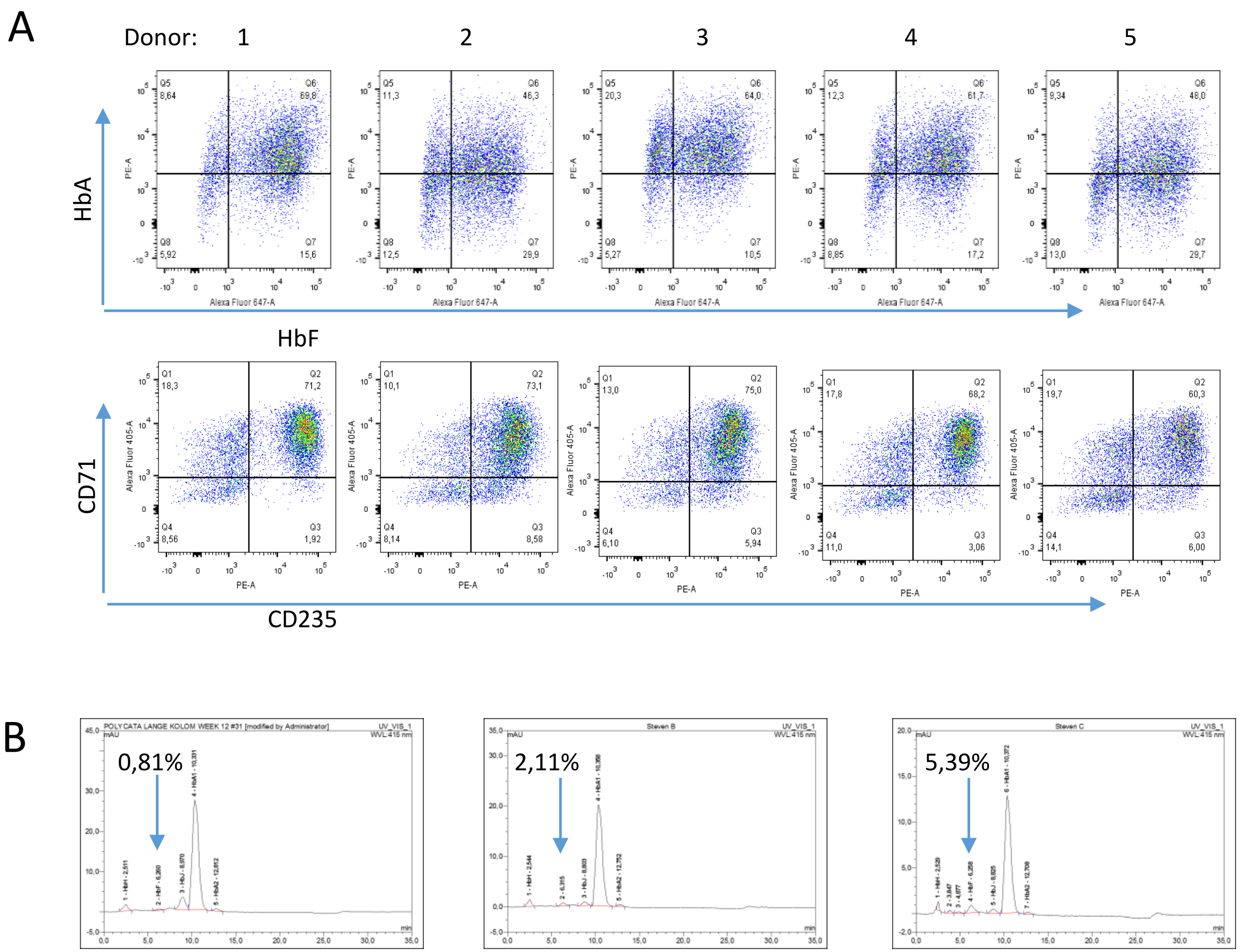

Supplemental figure 2

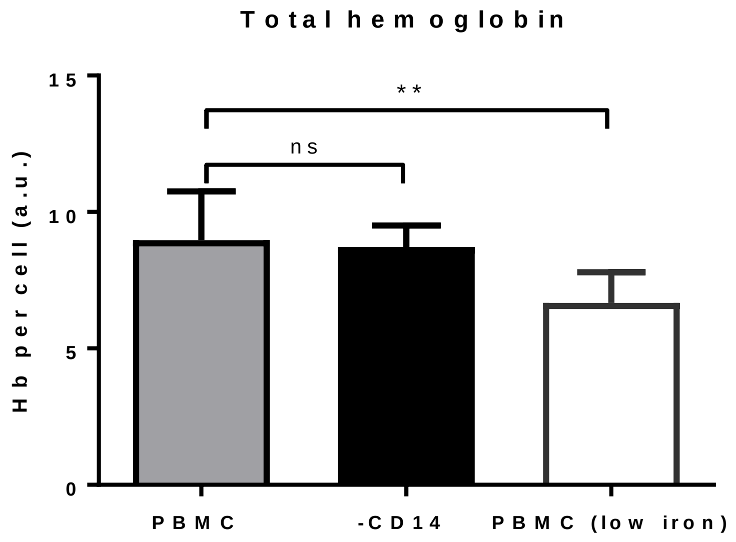

# Supplemental figure 3

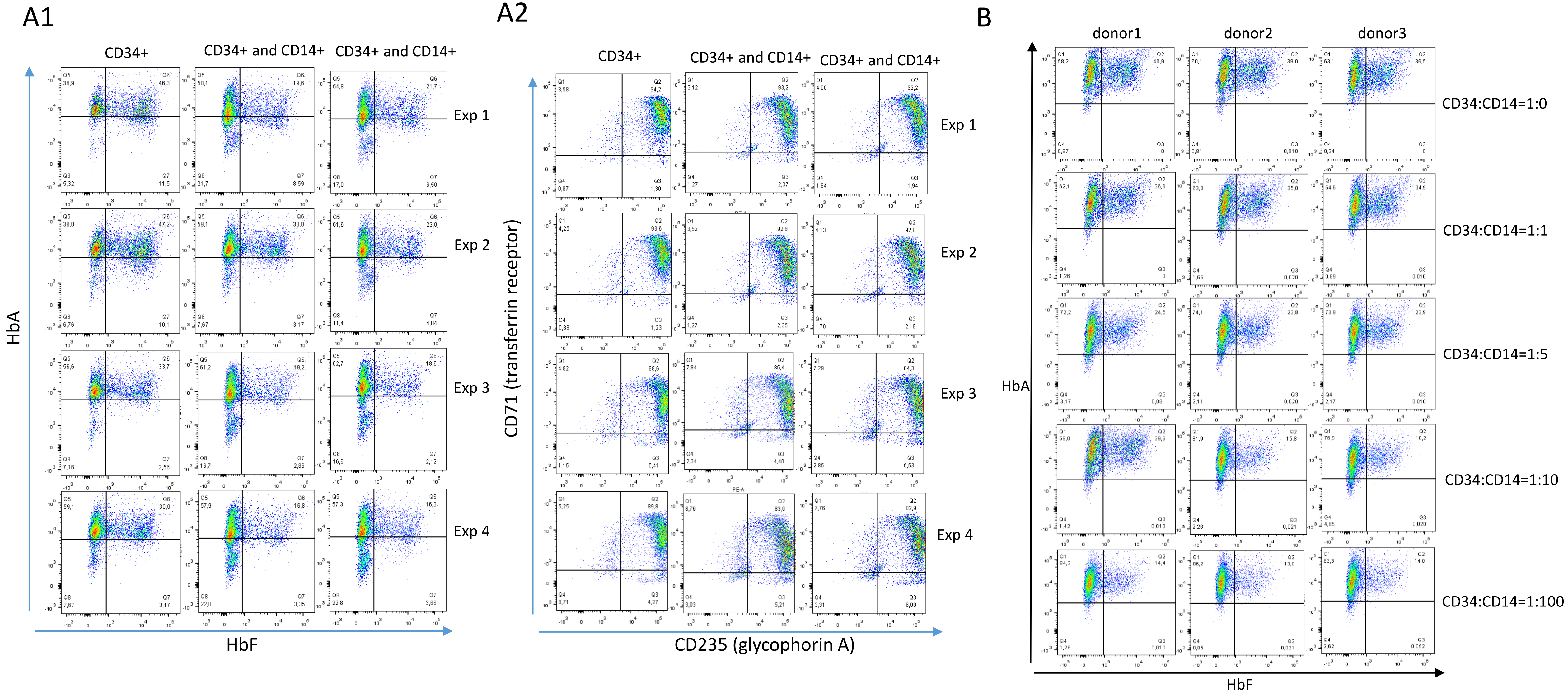

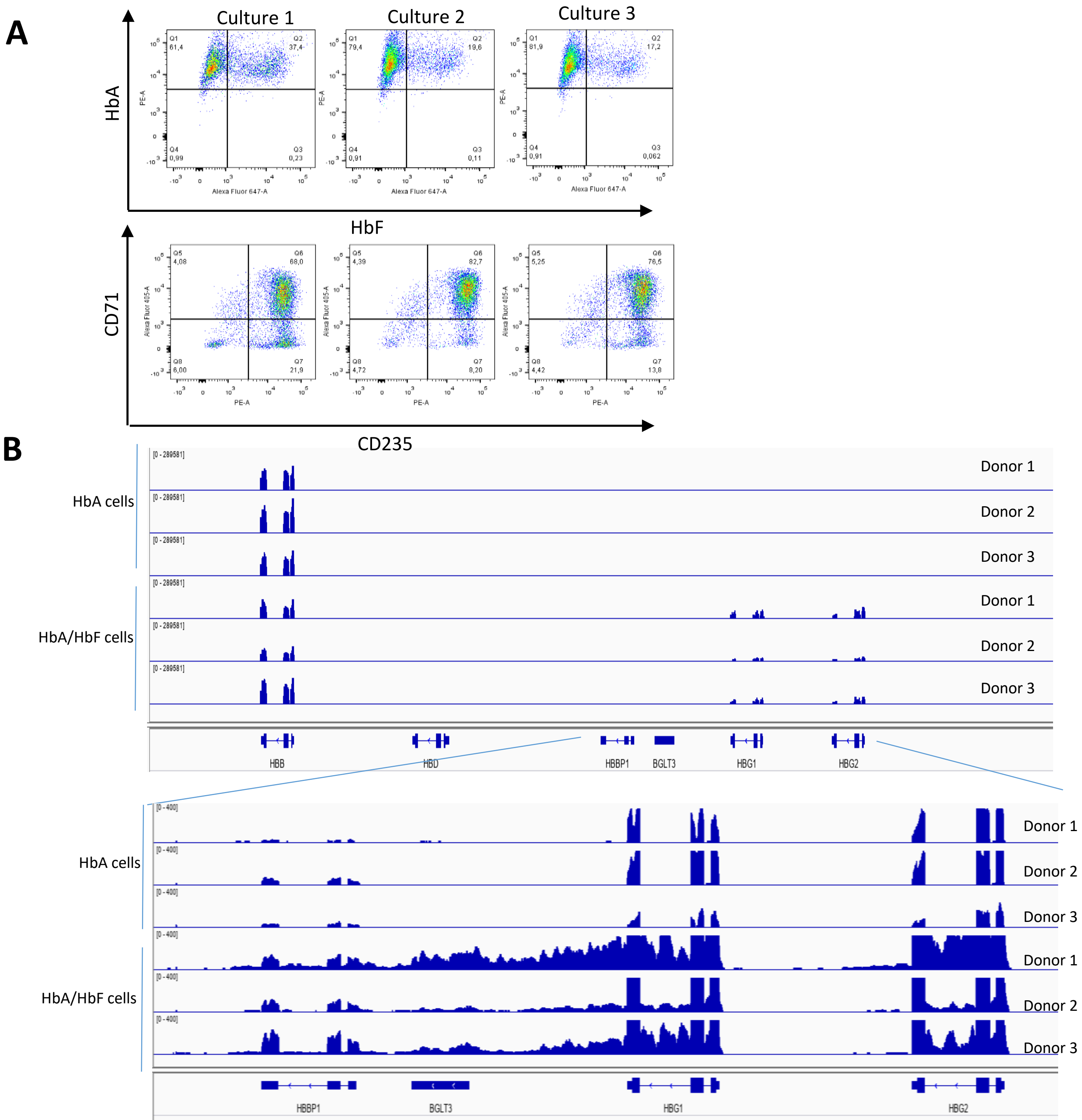


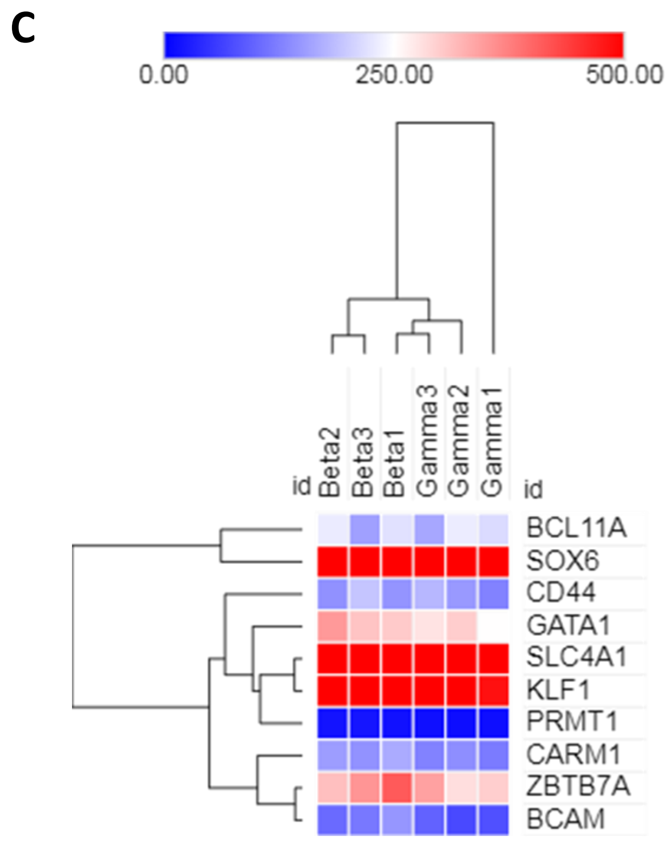


Supplemental figure 4
